# Mapping the Geographic Distribution of Tungiasis in Sub-Saharan Africa

**DOI:** 10.3390/tropicalmed5030122

**Published:** 2020-07-24

**Authors:** Mark A. Deka

**Affiliations:** Department of Geography, Texas State University; 601 University Drive, San Marcos, TX 78666, USA; mad214@txstate.edu

**Keywords:** tungiasis, GIS, disease mapping, neglected tropical disease, precision public health, medical geography

## Abstract

The geographic distribution of tungiasis is poorly understood, despite the frequent occurrence of the disease in marginalized populations of low socioeconomic status. To date, little work is available to define the geography of this neglected tropical disease (NTD). This exploratory study incorporated geostatistical modeling to map the suitability for tungiasis transmission in sub-Saharan Africa (SSA). In SSA, environmental suitability is predicted in 44 countries, including Angola, Nigeria, Ghana, Cameroon, Cote de Ivoire, Mali, Ethiopia, the Democratic Republic of the Congo, Kenya, Gabon, Uganda, Rwanda, Tanzania, Zambia, Zimbabwe, Madagascar, and South Africa. In total, an estimated 668 million people live in suitable areas, 46% (304 million) of which reside in East Africa. These evidence-based maps provide vital evidence of the potential geographic extent in SSA. They will help to guide disease control programs, inform policymakers, and raise awareness at the global level. Likewise, these results will hopefully provide decisionmakers with the pertinent information necessary to lessen morbidity and mortality in communities located in environmentally suitable areas.

## 1. Introduction

Tungiasis is a neglected tropical disease (NTD) caused by the parasitic flea *Tunga penetrans* (order: *Siphonaptera*, family: *Hectopsyllidae*). The disease has a widespread geographic distribution throughout the neotropical realm and sub-Saharan Africa (SSA). Ancient parasites like those from the genus *Tunga* have traditional roots in the Western Hemisphere among pre-Columbian Andean societies and were first described after the arrival of Columbus by Spanish chroniclers [1]. Documents from the early 20th century indicate that tungiasis caused severe morbidity in indigenous populations [2]. The spread of the ectoparasite to Africa was triggered in 1872 by the illegal dumping of sand ballast in Angola, having sailed from Rio de Janeiro, Brazil [2]. Prevalence rates in hyperendemic zones range from 21% to 83%, with a higher range found in Nigeria, Brazil, Trinidad, and Tobago [3,4,5]. As of 2009, tungiasis is endemic or potentially endemic in 89 countries worldwide [6]. The population estimated to be at risk of tungiasis in SSA is poorly documented. For example, in 2014, an estimated 1.4 million Kenyans suffered from the disease, and in 2011, there were 265 deaths, although it is likely that the number of fatalities is underreported [7]. 

**Figure 1:**
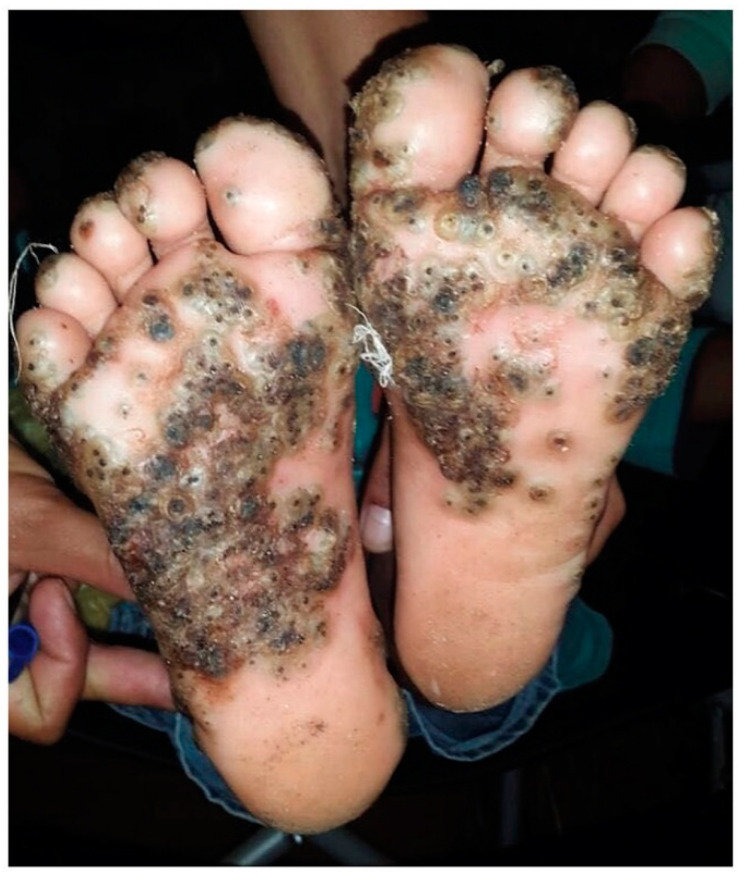
Severe tungiasis in the feet of a 10-year-old girl. Adapted from Barbosa and Barbosa [9]. License Number: 4832790723180.

To date, two species, *Tunga penetrans* and *Tunga trimamillata* are known to affect humans and domestic animals [8]. The disease develops when the female sand flea (*jigger flea or chigoe flea*), the smallest known flea in the world (< 1 mm), burrows into the host’s epidermis primarily in the hands, knees, heels, and soles [9,10] (Figure 1). This process causes acute clinical manifestations resulting in severe pain, itching, and a lesion at the site of penetration. The flea remains burrowed for a period of up to five weeks—during which, within one to two weeks, it increases its volume by a factor of roughly 2000–3000, before reaching a diameter of up to 1 cm [11,12]. Sand fleas have several unique physical features that give them the ability to burrow and remain buried in the epidermis. These include a well-developed laciniae and an epipharynx [13]. The entire parasite remains buried, with exception to the anus, the genital opening, and four pairs of stigmata [11]. 

Severe infestations can lead to the development of fissures, disfigurement, amputation, immobilization, and chronic lymphedema, as well as secondary infections like septicemia, tetanus, and toxic shock syndrome [14,15,16,17]. Individuals suffering from secondary medical conditions are at an increased risk of developing life-threatening complications [18]. Tungiasis is a disease of poverty that causes significant morbidity and mortality in children, the disabled, and the elderly [19]. The utilization of sharp instruments like needles or pins and even thorns to extract the fleas has been documented [15,19]. This practice raises the risk of contracting human immunodeficiency virus (HIV) and other blood-borne pathogens like Hepatitis B and C [18,20]. Ectopic infections of the genitals [21], eyelids [22], and tongue [23] have been documented. In resource-poor communities with low tetanus vaccination coverage, evidence points to an association between tungiasis and tetanus [16,24]. Individuals living with the disease often face social stigmatization and marginalization. In 2007, an estimated 800,000 Kenyans could not vote because of the inability to walk to voting stations due to disability from the disease [25]. 

Disease mapping is a field of spatial epidemiology involved in estimating the spatial patterns of disease risk to facilitate public health interventions [26]. Exploratory disease mapping provides insights, as opposed to diffusion, trends, or precise locational estimates [27]. Recent advances in geospatial technologies allow for greater flexibility in disease mapping. Data availability is a significant challenge to mapping the spatial distribution of parasitic, vector-borne, or zoonotic diseases. The availability of accurate epidemiological data, including precise spatially accurate occurrence and prevalence data, represents a significant barrier to progress. This challenge is no different with regards to tungiasis, a disease receiving little attention from policymakers, funding institutions, pharmaceutical companies, and the scientific community. To date, no systematic data on disease occurrence exist [28]. Likewise, control programs aimed at reducing severe morbidity are nonexistent [29]. 

The conceptual foundation of this study comes from two emerging fields, disease biogeography, and precision public health. Disease biogeography incorporates geospatial techniques, spatial epidemiology, and ecological niche modeling (ENM) to provide robust analytical and predictive analyses in the study of the geographic distribution of disease [30]. Examples of this application include mapping the potential risk of Mycetoma [31], Ebola virus disease (EVD) [32], anthrax (*Bacillus anthracis*) [33], Nipah virus (NiV) [34], and global hotspots of zoonotic diseases in mammals [35]. Precision public health integrates the geographic perspectives to pinpoint with greater accuracy the regions of elevated health risk at multiple scales of analyses. Literature examples of this include vaccine coverage [36], malaria [37], diarrhea [38], and child growth failure [39].

In this research, the goals were to map the geographic distribution of tungiasis in sub-Saharan Africa (SSA) by occurrences and remotely sensed environmental data. Despite representing a significant health burden, a thorough investigation of the geographic distribution of tungiasis in SSA is nonexistent. Thus, an exploratory analysis is necessary to (i) develop inductive models of environmental suitability and (ii) and to determine the total population living in areas of significant environmental heterogeneity where prioritization is needed. This study will be of interest to medical scientists, public health decisionmakers, policymakers, and other stakeholders interested in using geospatial techniques, especially regarding decisions on the equitable distribution of resources aimed at combating this disease.

## 2. Materials and Methods 

### 2.1. Study Area and Occurrence Records

**Figure 2:**
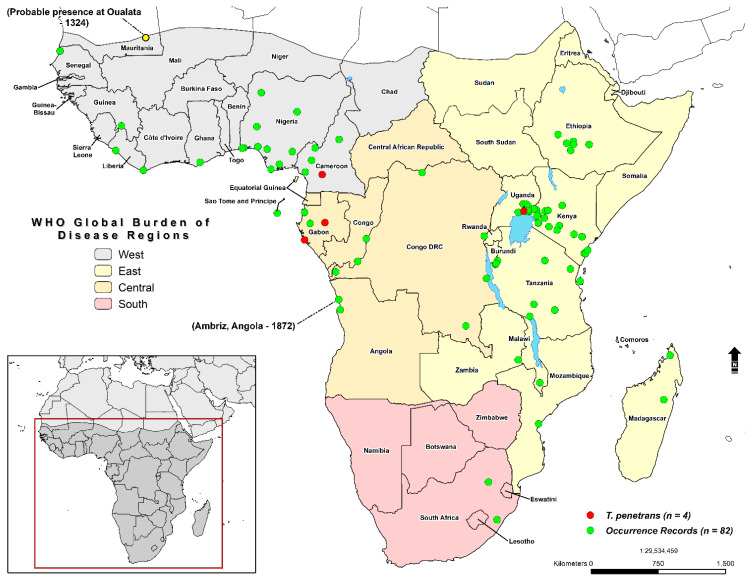
Model calibration region and study area defined by the WHO Global Burden of Disease Regions (GBD). Green dots correspond to occurrence locations (n = 86). The accepted historic arrival in Africa of *Tunga penetrans* in Ambriz, Angola (1872), and the probable presence at Oualata (1324) are highlighted.

The model calibration area (Figure 2) encompasses the whole of sub-Saharan Africa, a region defined broadly by the transition zone between the Saharan desert and the Sahel region. According to the World Bank, the total population of Sub-Saharan Africa in 2018 was 1,078,306,520 (https://data.worldbank.org/indicator/SP.POP.TOTL?locations=ZG). The study area is divided into designations from the WHO Global Burden of Disease Regions (GBD) [40]. These regional designations will serve as the basis for reporting the total population living in environmentally suitable areas. Tungiasis occurrences records (n = 86) (please see Supplementary Materials: Table S2) were assembled through a search of the literature from 1324–2019 [23,24,25,41,42,43,44,45,46,47,48,49,50,51,52,53,54,55,56,57,58,59,60,61,62,63,64,65,66,67,68,69,70,71,72,73,74,75,76,77,78,79,80,81,82,83,84,85,86,87,88,89,90,91,92,93,94,95,96,97]. The usually accepted arrival of *T. penetrans* in Africa is 1872, although evidence exists for its presence among a caravan traveling along the trans-Saharan trade route at Oualata (present-day Mauritania) in 1324 [97]. 

The date of this description is too early (548 years), assuming that *T. penetrans* was introduced to Africa after Columbus; however, it cannot be entirely dismissed based on historical accounts from Wiener [98], Hooton [99], and Hutton [75]. This record was not included in the modeling, though, due to these concerns. Briefly, the search for these records was conducted in the databases PubMed (https://pubmed.ncbi.nlm.nih.gov/) and Google Scholar (https://scholar.google.com/). Literature included cross-sectional surveys, monographs, and country-specific reports on the disease. The following search terms were used: "tungiasis", "tungiasis animals", and "tungiasis climate", as well as search terms for individual countries. For example, "tungiasis Kenya" or "tungiasis Nigeria". No time or article type limits were applied. Occurrence data were supplemented with Global Biodiversity Information Facility (GBIF) (https://www.gbif.org/) [41] records for *T. penetrans* in Gabon, Cameroon, and Uganda (n = 4) (GBIF.org (13 May 2020) GBIF Occurrence Download https://doi.org/10.15468/dl.xcpprz). Spatial information for each location (cities, towns, or villages) was collected manually from online geocoding services like Google Earth (https://earth.google.com/) and the Open Street Map project (openstreetmap.org). Geographic coordinates for these locations were standardized to decimal degrees to be displayed in a geographic coordinate system (WGS84). 

### 2.2. Description of Environmental Covariates

Data on the ecological determinants of tungiasis were obtained from a variety of remotely sensed datasets (Table 1). The geographic coordinates for the occurrence data (n = 86) were used to extract raster data estimates of (1) the bioclimatic variables; (2) enhanced vegetation index (EVI); (3) landcover type; (4) soil composition (sand, clay, and silt) and soil pH; (5) livestock densities; (6) distance to water bodies; (7) and the distribution of rural poor populations (person/square km). All datasets were resampled at a spatial resolution of 2.5 arcminutes (5-km) in ArcGIS desktop v10.7.1. [100].

Bioclimatic estimates were obtained from the ENVIREM (ENVIronmental Rasters for Ecological Modeling) [101] dataset at a 2.5-arcminute (5-km) resolution (https://envirem.github.io/). ENVIREM represents an alternative to the widely cited WorldClim database [101] and contains additional novel environmental information reflecting more specific underlying ecological and physiological processes [101]. ENVIREM is generated at the same resolution as WorldClim to facilitate integration and is processed for the present (1960–1990), mid-Holocene (~ 6000 years ago), and last glacial maximum (LGM) (~ 22,000 years ago) periods. Although these data are generated from the same underlying dataset as WorldClim, Title, and Bemmels [101] demonstrated marked improvements in the performances and projections of species distributions. Here, data for the current period (1960–1990) were obtained for the African continent in a GeoTiff format. Studies on the environmental factors influencing tungiasis are limited in scope; however, two previous studies have analyzed the off-host life stages of *T. penetrans* [13,102]. Linardi and colleagues [102] determined that, when examining *T. penetrans* larva, there was a difference in the air temperature and relative humidity between indoor and outdoor environments, with median values of 27.7 °C and 70.4% outdoors and 26.8 °C and 56.9% indoors, respectively. Furthermore, inside dwellings, larval *T. penetrans* could survive at air temperatures between 22.0–31.2 °C and relative humidity between 51.4–95.1% [102]. The evidence of tungiasis and soil characteristics has not been substantiated in previous research. However, Nagy and colleagues [13] found that 58% of larvae samples in a Brazilian study were located between 2–5 cm below the surface of the soil. Based on this information, edaphic variables: sand, clay, soil Ph, and silt content (5-cm depth, g/100 (w%)) were obtained at a 1-kilometer resolution from the International Soil Reference and Information Centre (ISRIC) (https://soilgrids.org/) at a 0–5-cm depth [103]. Local-level studies have documented an association between tungiasis and sandy, clay soils in Trinidad and Tobago [5], Brazil [104], and Kenya [51]. 

Enhanced vegetation index (EVI) (2001–2012) imagery was obtained from the United States Geological Survey Land Processes Distributed Active Archive Center (LP-DAAC) (http://LPDAAC.usgs.gov). Enhanced vegetation index (EVI) is an optimized vegetation index that increases the vegetation signal, with an emphasis on improved sensitivity in high biomass regions [105]. EVI has been identified as a proxy in previous studies of vector-borne [106] and parasitic diseases [107,108]. Complementing the EVI data were landcover coverages representing the predominant distribution of croplands, forests, grasslands, shrublands, and herbaceous vegetations (~1 km). These data were obtained from the Global Landcover Network Share database (GLC-Share) (2013) (http://www.fao.org/geonetwork/srv/en/main.home) managed by the Food and Agricultural Organization of the United Nations (FAO). The risk to humans is higher for individuals living with or in close contact with animals [109]. Thus, livestock densities were obtained from the Gridded Livestock of the World (v2.01) database at a 1~kilometer resolution from the Food and Agricultural Organization of the United Nations (FAO) (livestock.geo-wiki.org). These data include the densities of chickens [110], goats [24], and pigs [110]. Domestic animals serving as reservoirs for *T. penetrans* are numerous and include cats, dogs, chickens, goats, pigs, cattle, monkeys, and rats [7,15].

Socioeconomic and geographic factors, like with so many NTDs, are paramount to the persistence of these diseases. Tungiasis is highly prevalent among people living in extreme poverty in Latin America and SSA [15,18]. Epidemiological hotspots develop through the combination of pervasive poverty, behavioral attributes, and environmental risk factors [25]. With these facts in mind, estimates of the density of rural poor populations in SSA (2010) (person/square km) (5 km) (2.5-arcminute resolutions) were obtained from the Center for International Earth Science Information Network (CIESIN) (http://www.ciesin.org/) at Columbia University (New York, NY, USA). This data product combines several datasets: the 2005 Global Subnational Rates of Underweight Child Status, Global Rural-Urban Mapping Project (GRUMP), LandScan 2007 Global Population Database, the Demographic and Health Survey 2008, and the WHO Prevalence of Child Malnutrition database. Additionally, tens of millions of people in SSA suffer from a lack of access to adequate water resources or what is termed as "water poverty" [111]. The interface between water access and poverty is strongly interlinked [112,113]. Globally, it is estimated that hygiene and sanitation-related diseases account for 7% of the total disease burden and 19% of child mortality [114]. To represent this geographic determinant, the distances to water bodies (km) in SSA (2014) were obtained from the ESRI (Environmental Systems Research Institute) (https://www.arcgis.com/home/item.html?id=46cbfa5ac94743e4933b6896f1dcecfd) and were derived from the Global Lithological Map and a world landcover layer at a 250-meter resolution.

## 3. Data Analysis

### 3.1. Principal Component Analysis

A principal component analysis (PCA) was carried out on all sets of variables to reduce the overall multicollinearity and dimensionality [115,116]. The covariates were separated into five separate groups to calculate the principal components for each set using the cross-platform application, Niche Analyst (NicheA) [117]. For the ENVIREM data, the first eight PCs were retained, which represented 99% of the total variance. The first three PCs for soil (99.5%) and the first five PCs for landcover (99.9%) were retained, as were the first three for livestock densities (99.9%). Finally, for set five, two PCs (99.9%), corresponding to the distribution of rural poor populations in SSA and the distances to water bodies, were retained. Thus, the analysis was performed with a total of 21 environmental layers.

### 3.2. Ensemble ENM Approach

Ecological niche modeling (ENM) is a quantitative technique that links presence data with environmental variables, allowing for the development of a correlative model of environmental suitability. A "black box" approach, focusing on reconstructing the environmental conditions of disease persistence based on individual spatial information only, was implemented. The “black box” approach is advantageous for rare diseases where epidemiological information is scarce [118]. The ENM algorithms in this study were selected based on recommendations from Elith [119] and Eberhand [120]. These include generalized linear models (GLM) [121], generalized additive models (GAM) [121], generalized boosted regression models (GBM) [122], random forest (RF) [123], and maximum entropy (Maxent) [124]. Ensemble forecasting enabled the development of a more robust model that overcame the inherent uncertainties derived from each model [125]. 

The models were developed with the R programming language [126] package *biomod2* [127]. The model calibration specified that 80% of the data be assigned as a random sample, with the remaining 20% used for verifying the model accuracy using three metrics: the area under the curve (AUC) of the receiver operating characteristic (ROC) [128], the true skill statistic (TSS) [129], and Cohen’s Kappa (Heidke skill score) [130]. The algorithm parameters for the generalized linear models (GLM) specified a stepwise feature selection based on the Akaike Information Criterion (AIC); the generalized boosted regression models (GBM) featured 5000 maximum trees, a minimum of 10 tree terminal nodes, a learning rate of 0.01, and an interaction depth of 7. The generalized additive models (GAM) featured a binomial distribution and logit link function with a degree of smoothing set at 4; the random forest (RF) algorithm settings specified a node size of 5 and 500 maximum trees. Finally, the maximum entropy algorithm (Maxent) specified linear, quadratic, product, threshold, and hinge features and the number of iterations was increased from 500 to 5000 to ensure algorithm convergence. 

*Biomod2* requires a pseudo-absence generation when true absences are not available. Based on the small sample size, a conservative 1:1 ratio (86 pseudo-absences) between the pseudo-absences and occurrences was generated [131], with the pseudo-absence selection parameter “disk” that defined a minimal distance (250 km) to the occurrence points for selecting the pseudo-absence candidates. The pseudo-absences were randomly selected within the calibration area due to Maxent being a presence/background modeling technique. In total, ten replicates were specified, with a TSS evaluation metric threshold of > 0.50. The individual models were internally evaluated, with a subset of the data to determine the model performances and accuracies. Thus, only the models exceeding the evaluation metric cutoff of > 0.50 were retained. The final ensemble forecasts were estimated by weighting the mean of the TSS scores or, more specifically, the weighted sum of the predictions.

The coefficient of variation (CV) across all the predictions served as the measure of uncertainty (please see Map S1). The variable importance of the principal components was based on the decrease in accuracy and correlating the fitted data of the full models with the randomly permuted predictor values [132]. The subsequent uncertainty model was compared to the poverty estimates for the proportion of people living in poverty, as defined by the Multidimensional Poverty Index (MPI) [133] at a one-kilometer resolution for Kenya, Uganda, Tanzania, Nigeria, and Malawi. These estimates represent the proportion of people living on $1.25 a day. These data were obtained from the University of Southampton WorldPop project (https://www.worldpop.org/). 

### 3.3. Estimating the Population Living in Environmentally Suitable Areas

**Figure 3:**
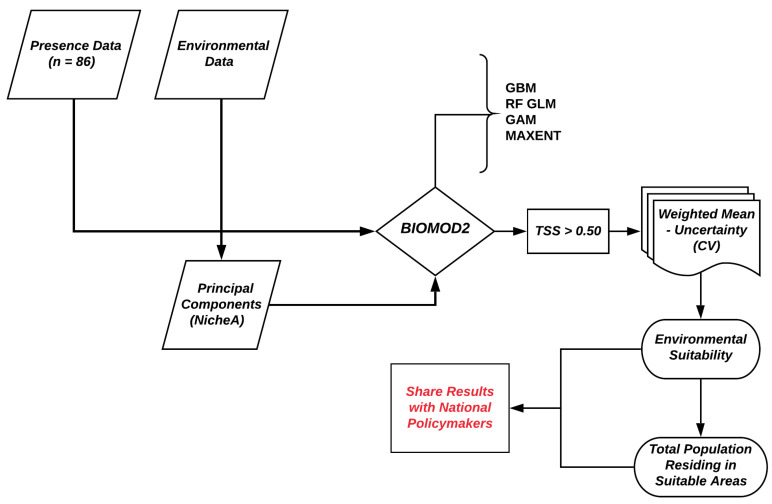
Workflow for tungiasis environmental suitability mapping.

The estimated population living in the areas of environmental suitability was determined by calculating a threshold value (i.e., ecological limits) that best represented the trade-off between the model sensitivity, specificity, and accuracy. The estimated number of individuals living in these areas in 2020 was calculated by overlaying this binary raster with the WorldPop (https://www.worldpop.org/) gridded population density surface. The calculated total population in these areas was quantified based on the number of individuals per grid cell based on the Global Rural-Urban Mapping Project (GRUMP) v1 (CIESIN) (https://sedac.ciesin.columbia.edu/data/collection/grump-v1) Columbia University (New York, NY, USA). The urban extent polygons delineated areas where the population density was greater than or equal to 1000 people per km^2^ based on the extent of nighttime light satellite imagery [134]. The areas with populations greater than or equal to 1000 were classified as urban, while cell values less than 1000 were assigned as nonurban. The resulting maps were created in ArcGIS desktop v10.7.1 [100]. The analytical strategy of this study is available in Figure 3.

### 3.4. Environmental Suitability in SSA

The compiled database (n = 86) of occurrences are located on landcover types that are primarily composed of croplands (27.7%), forests (22%), shrublands (13.3%), and grasslands (12.6%). Sand (49.13) and clay (33.20) contents were found to a higher degree when compared to silt (18.27), while the average soil pH was 5.7. The average headcount/km^2^ of livestock was highest among chickens (585.49), goats (32.05), and pigs (8.48). The average distance from each occurrence point to a water body was 64.25 km, and the average population density for rural poor per/km^2^ was 65.26. The average max temperature of the coldest month and the minimum temperature of the warmest month ranged from 26.4 °C (minimum: 17.5 °C, maximum: 33.8 °C) to 18.3 °C (minimum: 7.7 °C, maximum: 25.6 °C). The Thornthwaite aridity index average value of 55.19 (minimum: 4.67, maximum: 88.75) implied that occurrences were, on average, located in dry sub-humid environments. Dry sub-humid lands included grasslands and savannah biomes. The environmental suitability was further partitioned into climate zones based on the Koppen Climate Classification System [135]. Suitable environments were primarily found in tropical savanna (Aw), semi-arid (Bsh), humid subtropical (Cwa), tropical monsoon (Am), tropical rainforest (Af), and subtropical highland (Cwb) climates.

**Figure 4:**
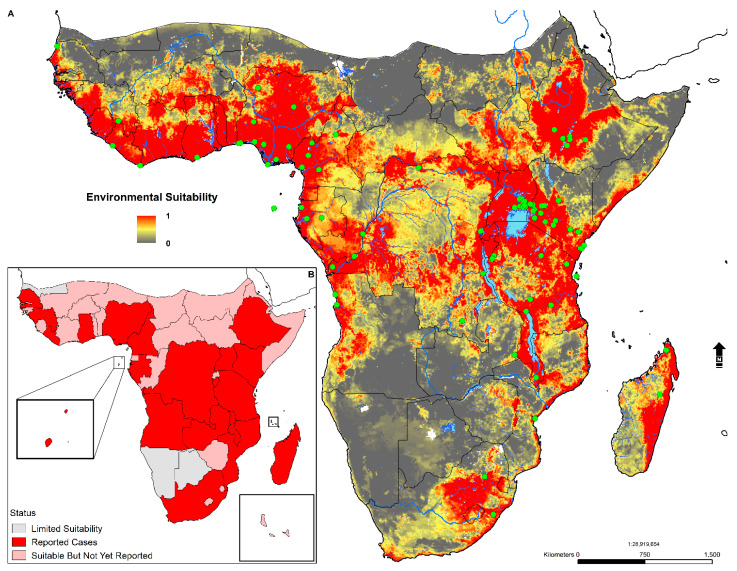
(**A**) Predicted distribution across sub-Saharan Africa (SSA). Probability of occurrence is provided on a scale from 0 (low probability) to 1 (high probability). Green dots correspond to occurrence locations (n = 86). (**B**) Status of occurrence reporting by country in SSA based on the weighted mean suitability model. Insets correspond to São Tomé and Príncipe and Comoros.

The probability of occurrence based on the weighted mean output is presented in Figure 4. High suitability was predicted in 44 countries in SSA, along with areas surrounding the African Great Lakes (Kyoga, Albert, Edward, Victoria, Kivu, Tanganyika, Rukwa, Mweru, and Malawi); Eastern and Central Madagascar; the Ethiopian Highlands; Eastern South Africa; Nigeria; Cameroon; Tanzania; and Western Angola. Suitability was also present in South Sudan, the Central African Republic, Cote de Ivoire, Guinea-Bissau, Ghana, Liberia, Sierra Leone, and The Gambia. Additionally, environmental signals were captured in Burkina Faso, Zimbabwe, Mali, Somalia, and Gabon. Most of Southwestern Africa (Namibia and Botswana) and Southern Mauritania was not predicted to be suitable. Please see the Supplementary Materials for the map of the model uncertainty (CV) (Map S2).

**Figure 5:**
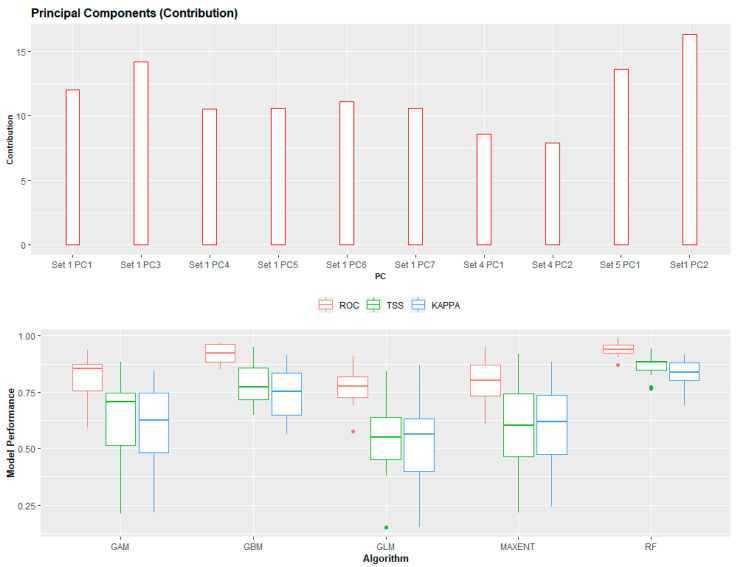
Variable contributions (**top**) and predictive algorithm performances (**bottom**). A. PC: principal component contribution. B. ROC: receiver operating characteristic, KAPPA: Cohen’s Kappa, and TSS: true skill statistic.

Validation statistics (Figure 5) for the selected algorithms indicated good-strong performances. Overall, the random forest (RF) (AUC: 0.94; TSS: 0.86; KAPPA: 0.83), generalized boosted regression (GBM) (AUC: 0.86; TSS: 0.70; KAPPA: 0.68), and generalized linear models (GLM) (AUC: 0.83; TSS: 0.65; KAPPA: 0.63) outperformed both the GAM and Maxent (see Supplementary Materials: Table S1). Variable contributions for the principal components strongly favored bioclimatic factors (Set1 PC2—16.28, Set1 PC3—14.17, Set1 PC1—12, Set1 PC6—11.13, Set1 PC7—10.59, Set1 PC5—10.58, and Set1 PC4—10.54); livestock densities (Set4 PC1—8.6 and Set4 PC2—7.9); the distance to water and the distribution of rural poor populations (Set5 PC1—13.65). An environmental suitability threshold of 0.438 provided the best estimate when measuring the relationships between the weighted mean model sensitivity, specificity, and accuracy. The population living in environmentally suitable areas is estimated at 668 million (Table 2) or 62% of the total population of SSA. The largest proportion of the population is located in East Africa (46%), followed by West Africa (40%) and Central Africa (9%). East Africa has the largest total population living in nonurban areas (217,725,514), while West Africa has the largest total population living in urban areas (148,452,658). The total area of environmentally suitable areas (threshold > 0.438) totaled 8,110,107 sq. kilometers or 3,131,329 sq. miles.

## 4. Discussion

**Figure 6:**
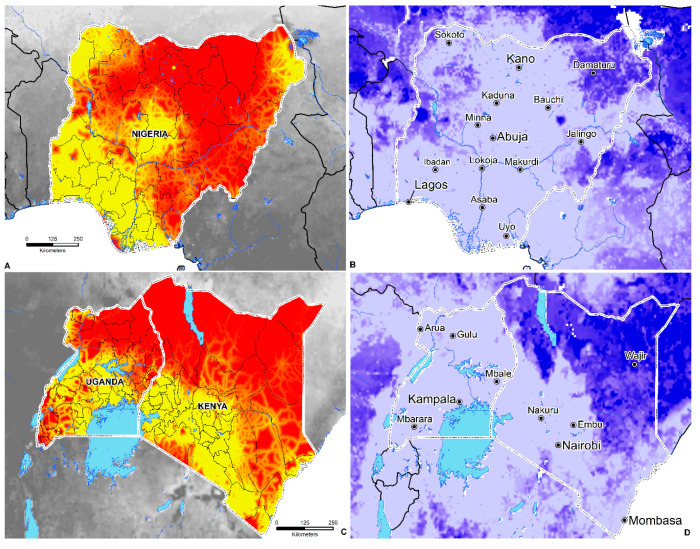
(**A,C**) The proportion of people living on $1.25 a day in Nigeria, Uganda, and Kenya. Red corresponds to a higher proportion of the population living on $1.25 a day; yellow represents a lower proportion of the population living on $1.25 a day. (**B,D**) The coefficient of variation (CV) or measure of model uncertainty. Lighter colors = less uncertainty; darker colors = increased uncertainty.

**Figure 7:**
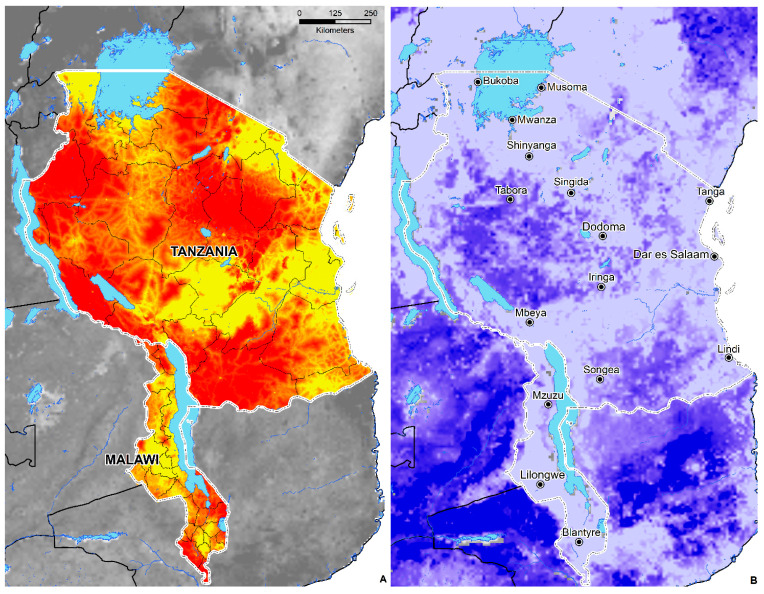
(**A**) The proportion of people living on $1.25 a day in Tanzania and Malawi. Red corresponds to a higher proportion of the population living on $1.25 a day; yellow represents a lower proportion of the population living on $1.25 a day. (**B**) The coefficient of variation (CV) or measure of model uncertainty. Lighter colors = less uncertainty; darker colors = increased uncertainty.

Since the first description of Tungiasis in 1526 among members of Columbus’ crew of the Santa Maria [2] to the subsequent expansion of the parasitosis across Africa in the 19th century, the disease has become unfortunately commonplace among underprivileged communities facing extreme poverty and underdevelopment. This study is the first to examine tungiasis at a continental scale and contributes to the recent literature that examined the disease from a geographic perspective [15,51]. An ecological niche modeling (ENM) approach was applied to understand better the relationship between the distribution of occurrence locations and environmental covariates to develop a broad-scale predictive map of suitability across SSA. These findings are useful for measuring the degree of spatial heterogeneity and to estimate the total human population living in environmentally suitable areas. Mapping the geographic distribution of tungiasis in SSA represents a positive step towards greater awareness in not only Africa but much of the Western world, where the disease, its impact on human life, and the ectoparasite *T. penetrans* are unheard of by many. Likewise, model-based guidance is useful for identifying environmentally suitable areas and the potential allocation of resources. NTDs are intimately linked to poverty [136] and thrive in areas with poor sanitation, lack of access to clean water, and among people who live near infected disease vectors and animals [137]. These predictions can further guide surveillance and facilitate dialogue between public health interests located in areas predicted to be suitable, especially those of low socioeconomic status (Figure 6 and Figure 7). 

Environmental factors play a vital role in governing the distribution of disease vectors, pathogenic microorganisms, and/or the persistence and transmission of the disease [138]. These results illustrate that bioclimatic factors, the density of livestock, the distance to water, and the distribution of rural poor populations were significant contributors to the potential geographic distribution. Since no previous work has analyzed tungiasis at a broad, continental scale, it is admittedly challenging to compare the results from this study to the previous literature. The broad geography of the occurrence data should be considered, as this clearly reflects the magnitude of the problem for potentially hundreds of millions of people living in suitable areas throughout SSA. This study predicted 44 countries as being suitable for tungiasis. The potential distribution of suitable environments in SSA corresponds to countries with confirmed cases, and these include Angola, Nigeria, Ghana, Cameroon, Ethiopia, the Democratic Republic of the Congo, Kenya, Gabon, Uganda, Tanzania, Zambia, Madagascar, and South Africa. Countries predicted as environmentally suitable but without documented cases include Burkina Faso, Cote de Ivoire, Mali, Chad, Niger, the Central African Republic, Senegal, Sudan, South Sudan, Somalia, and Zimbabwe. Countries with limited suitability are Botswana, Namibia, and Mauritania. These environmentally suitable areas are located predominately within tropical savanna (Aw), semi-arid (Bsh), humid subtropical (Cwa), tropical monsoon (Am), tropical rainforest (Af), and subtropical highland (Cwb) climates. 

Geographically, the natural biomes found in these classifications range from grasslands in South Africa, Ethiopia, Zimbabwe, and Zambia to savannas and shrubland biomes in Angola, Tanzania, Kenya, Uganda, South Sudan, and Nigeria. Additionally, tropical rainforests and woodlands stretching from Liberia, Southern Cote d’Ivoire, Nigeria, Western Cameroon, and Eastern Madagascar were highlighted. The population living in environmentally suitable areas is estimated at 668 million (2020), with the majority of these individuals residing in WHO GBD east (46%) and west (40%) regions, which, combined with GBD central and south regions, represent approximately 62% of the total population of SSA. NTDs primarily inflict suffering in poor rural agricultural communities, particularly in regions with subsistence agriculture [139]. East Africa is home to the largest population living in environmentally suitable areas, with an estimated 217 million residents. East Africa is noted as being the most isolated region on the continent, with an estimated 74.4% of the population residing in rural areas [140]. Given the high degree of local and individual heterogeneity within the geographic regions or enumeration units, an effective policy design requires detailed knowledge of the spatial demographics of populations residing in environmentally suitable areas. Disease transmission, because of the clustered nature of the human population, tends to trend towards being spatially focal and heterogeneous. This study contributes to the incorporation of spatial demographic and epidemiological data in disease mapping. Previous efforts in this realm include estimating the burden of malaria in children under five years old [141] and the risk of schistosomiasis [142] and soil-transmitted helminths [143].

This study was grounded in a conceptual foundation of precision public health [144] and disease biogeography [30]; both are rapidly evolving fields that play a critical role in mapping the disease transmission risk in public and animal health. Due to the complex web of causation associated with tungiasis prevalence and hyperendemicity, a multidisciplinary approach incorporating both conceptual frameworks with the expert advice of medical doctors, entomologists, ecologists, parasitologists, and public health officials has the potential to lessen the burden of the disease considerably in geographies of high suitability. Gaining greater insights at more granular resolutions to the inherent risks to human populations is a central theme to frameworks like precision public health, which, at its core, uses the best available data to target more efficient interventions to those most in need [145]. Understanding and anticipating “where” an outbreak may take place or “where” resources need to be allocated to have the most impact are valuable tools for public health interventions.

Although the techniques described in this study offered useful information, there were limitations associated with this research. First, the data on tungiasis in SSA is scarce in comparison to other NTDs on the continent. Therefore, the occurrence records collected in this study spanned many centuries and were compiled from a wide variety of literature sources; thus, there is some inherent uncertainty associated with these data. Some of the oldest records included in the final dataset, particularly the description of apparent jigger infestations at Oualata in 1324, need additional investigation. Though it may never be confirmed, this record brought to light by Lugard [97] raises further questions about the existence of the ectoparasite on the African continent hundreds of years before the historically accepted arrival data of 1872. Other records in this study only include the district name or relative location within these enumeration units.

Second, the suitability model does not measure tungiasis prevalence or incidence. The correlative model characterizes the similarities between the occurrence locations based on environmental covariates and does not estimate the magnitude or confirm their presence. Moreover, the development of inductive models can lead to difficulties in extracting causality. Tungiasis represents a disease with a complex web of causation due to the broad spectrum of domestic and wild animal hosts and prevalence in resource-poor communities. It is crucial to appreciate that these maps delineate areas that are environmentally suitable for tungiasis, and they do not predict the likelihood of the disease in humans and animals and the subsequent human, domestic, and sylvatic cycles. Third, another limitation of these geostatistical estimates is that the entire transmission cycle can occur indoors without the involvement of a zoonotic reservoir [146]. As demonstrated by Linardi and colleagues [102], in resource-poor communities and urban-rural environments, *T. penetrans* can develop from eggs to adults either indoors or in “indoor-outdoor” habitats. Future mapping efforts at the local scale would benefit from the incorporation of risk factors associated with tungiasis, as well as data representing the status of poor or improved housing conditions.

## 5. Conclusions

In conclusion, this work highlights, for the first time, the potential geographic distribution of tungiasis in sub-Saharan Africa. This work has identified areas potentially suitable for tungiasis, which need priority for future mapping endeavors and interventions. Significant findings from this research:

- An estimated 668 million individuals live in environmentally suitable areas: 304 million in WHO GBD East Africa and 263 million in WHO GBD West Africa.
- Geographically, environmental suitability is broad and diverse, ranging from tropical savanna (Aw), semi-arid (Bsh), humid subtropical (Cwa), tropical monsoon (Am), tropical rainforest (Af), and subtropical highland (Cwb) climates.
- Environmental suitability is predicted in 44 countries; these include Angola, Nigeria, Ghana, Cameroon, Cote de Ivoire, Mali, South Sudan, Sudan, Somalia, Ethiopia, the Democratic Republic of the Congo, Kenya, Gabon, Central African Republic, Uganda, Rwanda, Tanzania, Zambia, Zimbabwe, Madagascar, Mozambique, and South Africa.
- The total area of environmental suitability (weighted mean threshold > 0.438) is 8,110,107 sq. kilometers or 3,131,329 sq. miles.

Future research would benefit from transdisciplinary collaborations in the development of the first suitability maps for Latin America and improved estimates for SSA. It is imperative to increase the scientific knowledge about the disease and advance greater awareness. Geographically targeted surveillance activities in epidemiological hotspots are critical to managing tungiasis. These maps provide a foundational and baseline assessment that is an essential prerequisite for data-driven control programs. 

## Figures and Tables

**Table 1:** Environmental covariates. SSA: sub-Saharan Africa, ENVIREM: ENVIronmental Rasters for Ecological Modeling, ISRIC: International Soil Reference and Information Centre, GLC-Share: Global Landcover Network Share database, FAO: Food and Agricultural Organization of the United Nations, ESRI: Environmental Systems Research Institute, and CIESIN: Center for International Earth Science Information Network.

Covariates	Spatial Resolution	Data Source	Units	Average
**Set** **1**				
Annual Potential Evapotranspiration (PET)	~5 km	ENVIREM	mm/year	1625.14
Thornthwaite Aridity Index	~5 km	ENVIREM		55.19
Climatic Moisture Index	~5 km	ENVIREM		–0.13
Continentality	~5 km	ENVIREM	°C	3.55
Emberger’s Q	~5 km	ENVIREM		352.04
Growing Degree Days Greater than 0 °C	~5 km	ENVIREM		97,927.53
Growing Degree Days Greater than 5 °C	~5 km	ENVIREM		98,456.92
Max Temp Coldest Month	~5 km	ENVIREM	°C × 10	26.4
Min Temp Warmest Month	~5 km	ENVIREM	°C × 10	18.3
Month Count with Temp Greater than 10 °C	~5 km	ENVIREM	months	12
PET Coldest Quarter	~5 km	ENVIREM	mm/month	120.57
PET Driest Quarter	~5 km	ENVIREM	mm/month	133.28
PET Seasonality	~5 km	ENVIREM	mm/month	1428.88
PET Warmest Quarter	~5 km	ENVIREM	mm/month	150.87
PET Wettest Quarter	~5 km	ENVIREM	mm/month	133.78
Thermicity Index	~5 km	ENVIREM	°C	584.62
**Set** **2**				
Sand (0–5 cm)	~1 km	ISRIC	g/100 (w%)	49.13
Silt (0–5 cm)	~1 km	ISRIC	g/100 (w%)	18.27
Clay (0–5 cm)	~1 km	ISRIC	g/100 (w%)	33.20
Soil pH (0–5 cm)	~1 km	ISRIC	g/100 (w%)	5.7
**Set 3**				
Enhanced Vegetation Index (EVI) 16-day composites (2001–2012)	~1 km	NASA		0. 36
Cropland (2013)	~1 km	GLC-Share	%	29.76
Herbaceous (2013)	~1 km	GLC-Share	%	1.95
Grassland (2013)	~1 km	GLC-Share	%	12.66
Shrubland (2013)	~1 km	GLC-Share	%	13.3
Tree Covered Area (2013)	~1 km	GLC-Share	%	22
**Set 4**				
Goats Density (2014)	~1 km	FAO	head/km^2^	32.05
Pig Density (2014)	~1 km	FAO	head/km^2^	8.48
Chicken Density (2014)	~1 km	FAO	head/km^2^	585.49
**Set 5**				
Distance to Water (2014)	250 meters	ESRI	km	64.25
Rural Poverty in SSA (2010)	~5 km	CIESIN	person/km^2^	65.26

**Covariates**

**Spatial Resolution**

**Data Source**

**Units**

**Average**

**Table 2:** The total population living in environmentally suitable areas in SSA (2020). WHO GBD: WHO Global Burden of Disease Regions.

WHO GBD Region	Total Population	% of Total	Urban (>1000)	Nonurban (<1000)
West	263,954,435	40%	148,452,658	115,475,777
East	304,529,659	46%	86,753,145	217,725,514
Central	61,693,453	9%	22,428,932	39,261,521
South	37,945,399	6%	17,213,237	20,728,162
Total	668,122,946		274,847,972	393,190,974

## Supplementary Materials

The following are available online at https://www.mdpi.com/2414-6366/5/3/122/s1: Table S1: Modeling algorithm predictive performance (ROC, TSS, and KAPPA). Map S1: Model Uncertainty Map (Coefficient of Variation). Map S2: Binary (presence/absence)—weighted mean threshold: 0.438. Table S2: Tungiasis occurrence locations in SSA (n = 87).
