# Supplementary material for: Mapping the Geographic Distribution of Tungiasis in Sub-Saharan Africa
Source: Trop Med Infect Dis. 2020 Jul 24;5(3):122. doi: 10.3390/tropicalmed5030122 (PMC7558156; doi:10.3390/tropicalmed5030122)
Supplement: Supplementary file 1 [file tropicalmed-05-00122-s001.pdf]

Tropical Medicine and Infectious Disease

Supplementary Materials

SUPPORTING INFORMATION for: Mapping the Geographic Distribution of Tungiasis in Sub-Saharan Africa

Table of Contents

Table S1: Modeling algorithm predictive performance

Map S1: Model Uncertainty Map (Coefficient of Variation)

Map S2: Binary (presence/absence) – weighted mean threshold: 0.438

Table S2: Tungiasis occurrence locations in SSA (n = 87)

Table 1. Weighted mean validation indicators (AUC, TSS, KAPPA) for the tested modeling approaches: ROC: the area under the receiver operating characteristic (ROC) curve, TSS: true skill statistic, Cohen's Kappa (Heidke skill score).

GAM			GBM			GLM			MAXENT			RF		
**ROC**	**TSS**	**KAPPA**	**ROC**	**TSS**	**KAPPA**	**ROC**	**TSS**	**KAPPA**	**ROC**	**TSS**	**KAPPA**	**ROC**	**TSS**	**KAPPA**
0.81	0.63	0.61	0.86	0.70	0.68	0.83	0.65	0.63	0.83	0.64	0.62	0.94	0.86	0.83

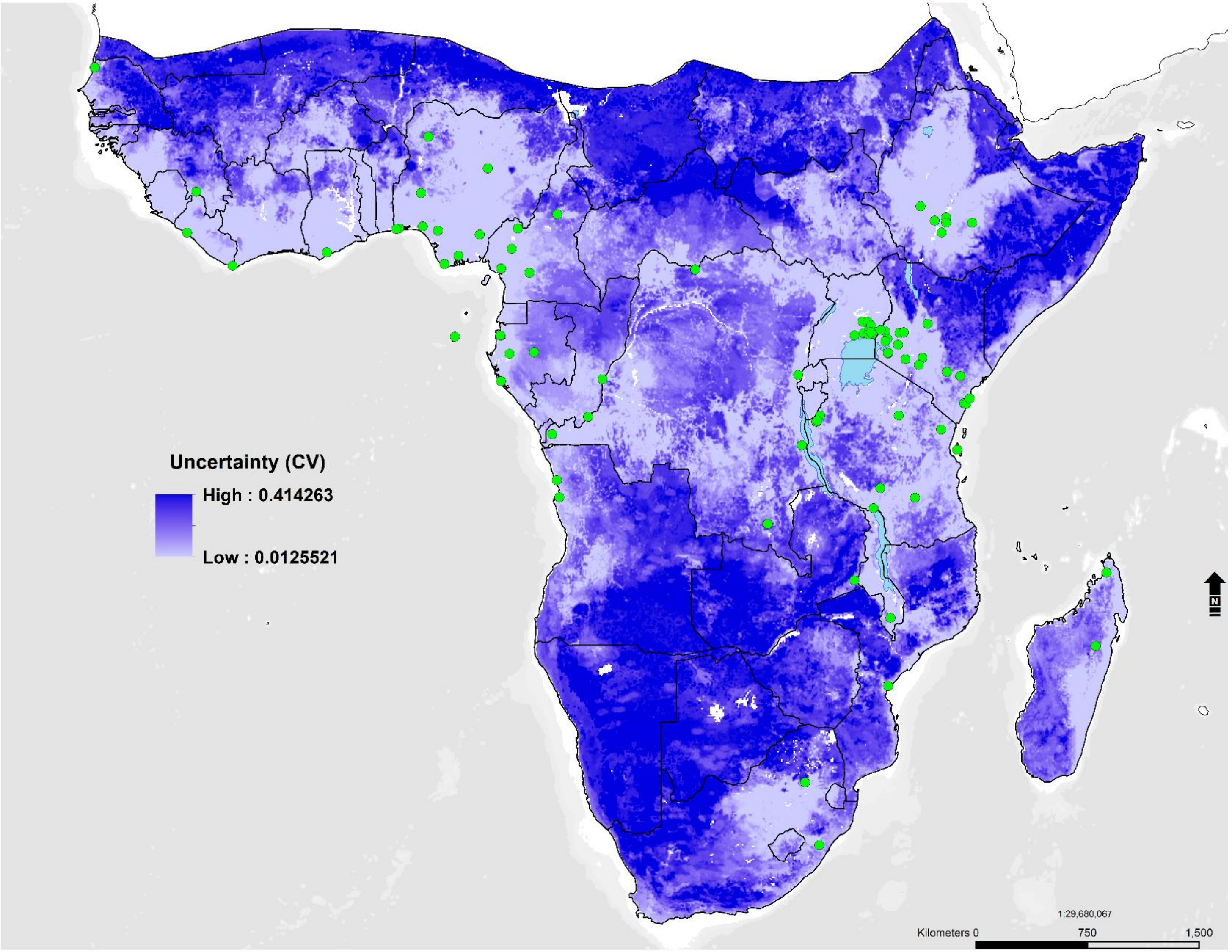

Map S1: A. Uncertainty (Coefficient of Variation).

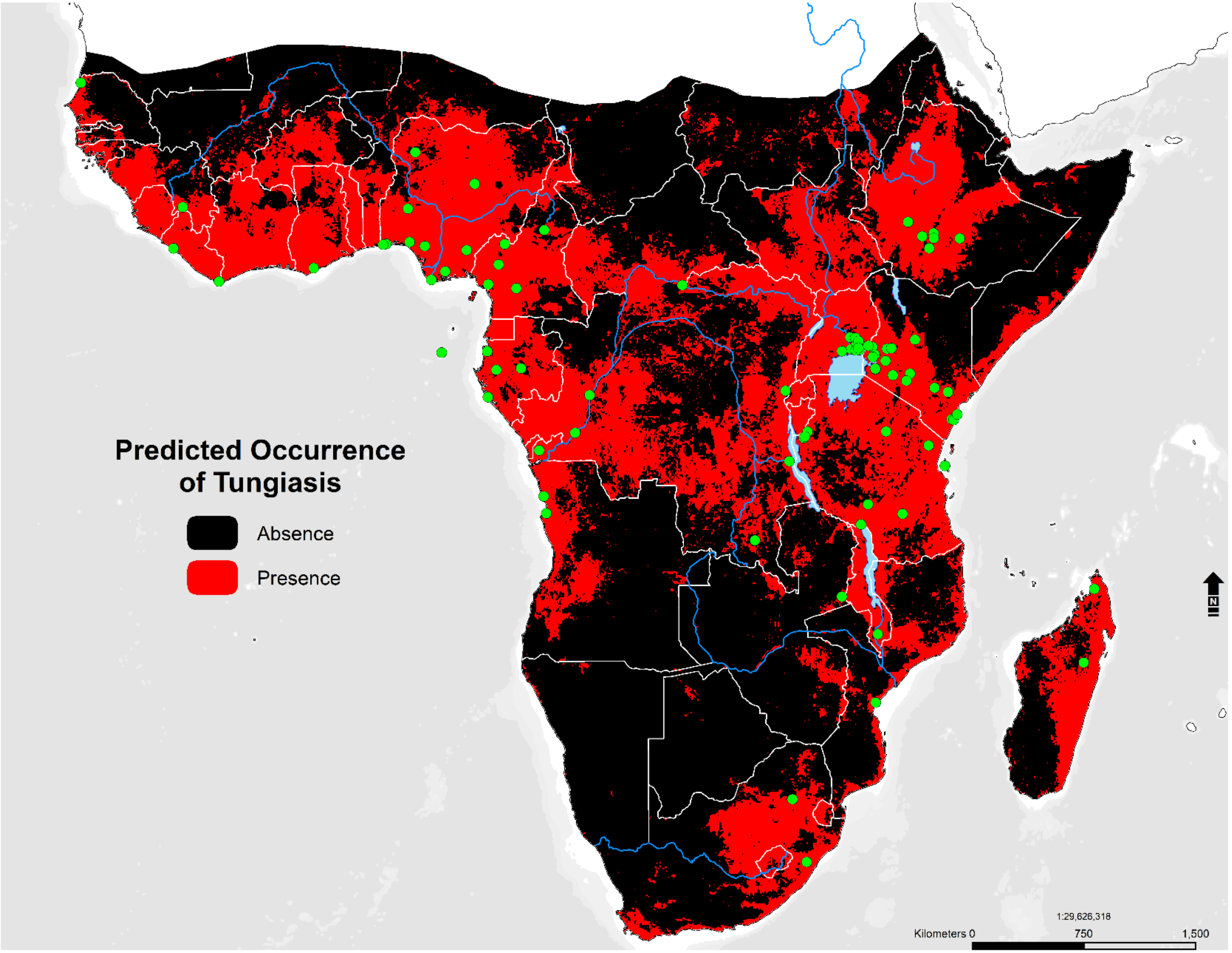

Map S2: Binary (presence/absence) – weighted mean threshold: 0.438.

Table 2. Tungiasis occurrence locations in SSA (n = 87).

Longitude	Latitude	Country	Source	Summary of Findings
33.1962	0.43936	Uganda	GBIF.org (13 May 2020) GBIF Occurrence Download https://doi.org/10.15468/dl.xcpprz	Human Observation
9.58941	-2.2466	Gabon	GBIF.org (13 May 2020) GBIF Occurrence Download https://doi.org/10.15468/dl.xcpprz	Human Observation
11.79	-0.6204	Gabon	GBIF.org (13 May 2020) GBIF Occurrence Download https://doi.org/10.15468/dl.xcpprz	Preserved Specimen
11.5199	3.89846	Cameroon	GBIF.org (13 May 2020) GBIF Occurrence Download https://doi.org/10.15468/dl.xcpprz	Preserved Specimen
8.87296	9.88455	Nigeria	Ames, C.G. Gazetteers of the Northern Provinces of Nigeria, Volume IV: The Highland Chieftaincies (Plateau Province); 1934.	Geographical Dictionary
8.32397	6.09058	Nigeria	Partridge, C. Cross River natives: being some notes on the primitive pagans of Obubura Hill district, southern Nigeria, including a description of the circles of upright sculptured stones on the left bank of the Aweyong River.; London, 1905.	Ethnological study
6.02788	4.4246	Nigeria	Bindloss, H. In the Niger country; W. Blackwood and sons, 1898.	A non-fiction account of travels to Africa
36.8364	7.67878	Ethiopia	Ramaswamy, V. Assessment of socioeconomic status and the prevalence of Tungiasis in Jimma and Wolaita Sodo, Ethiopia. Int J Intg Med Sci 2016, 3, 211–15.	High prevalence of tungiasis could be significantly associated with the low income, poor housing, and low standard living conditions, and poor health care behavior of the population (p-value <0.001).
38.488	7.03926	Ethiopia	Tadele, H. Clinical profile and outcome of pediatrics tetanus: the experience of a tertiary hospital in Ethiopia. Ethiopian journal of health sciences 2017, 27, 559–564.	Trauma was common above 5 years of age, and tungiasis was documented in 5 cases. All patients had generalized tetanus.
38.2098	6.18401	Ethiopia	Walker, S.L.; Lebas, E.; De Sario, V.; Deyasso, Z.; Doni, S.N.; Marks, M.; Roberts, C.H.; Lambert, S.M. The prevalence and association with health-related quality of life of tungiasis and scabies in schoolchildren in southern Ethiopia. PLoS neglected	There was a high burden of skin disease amongst this cohort with more than 40% having an ectodermal parasitic skin disease. The majority of these were due to tungiasis.
33.1204	1.10608	Uganda	Sentongo, E.; Wabinga, H. Tungiasis presenting as a soft tissue oral lesion. BMC oral health 2014, 14, 112.	Tungiasis of the tongue was diagnosed after microscopic examination.
33.5487	0.74916	Uganda	Sentongo, E.; Wabinga, H. Tungiasis presenting as a soft tissue oral lesion. BMC oral health 2014, 14, 112.	Tungiasis of the tongue was diagnosed after microscopic examination.
33.4552	1.08454	Uganda	Sentongo, E.; Wabinga, H. Tungiasis presenting as a soft tissue oral lesion. BMC oral health 2014, 14, 112.	Tungiasis of the tongue was diagnosed after microscopic examination.
35.5098	0.46682	Kenya	Chelimo, J.J. Risk factors associated with jigger infestation in Kitany location, Keiyo Marakwet county, Kenya. 2015.	There was little difference in prevalence between children 5 to 9 years (16.0%) and adults (15%). Infestation was highest in the elderly (40.1%).
34.5604	0.56649	Kenya	Mørkve, Å. " Getting rid of the plague": jiggers removal program in Bungoma, Kenya. Community and health workers perspectives on tungiasis in a high prevalence area. 2013.	Tungiasis is a debilitating problem affecting individuals' households and increasing the vicious circle of poverty.
34.2847	0.63625	Kenya	Ruttoh, S.K.; Ochieng'Omondi, D.; Wanyama, N.I. Tunga penetrans A Silent Setback to Development in Kenya. Journal of Environmental Science and Engineering. B 2012, 1.	As a country, the Jigger menace is frustrating Kenya's attempts to achieve the Millennium Development Goal on poverty reduction by the year 2015, as well as reducing the pace of national development.
33.7465	0.39985	Uganda	Mutebi, F.; Krücken, J.; Feldmeier, H.; Waiswa, C.; Mencke, N.; Sentongo, E.; von Samson-Himmelstjerna, G. Animal reservoirs of zoonotic tungiasis in endemic rural villages of Uganda. PLoS Negl Trop Dis 2015, 9, e0004126.	Animal and human tungiasis were closely associated and pigs were identified as the most important animal hosts of *T. penetrans*.

32.5922	0.32086	Uganda	Sentongo, E.; Wabinga, H. Tungiasis presenting as a soft tissue oral lesion. BMC oral health 2014, 14, 112.	Tungiasis of the tongue was diagnosed after microscopic examination.
33.4916	0.37796	Uganda	Wafula, S.T.; Ssemugabo, C.; Namuhani, N.; Musoke, D.; Ssempebwa, J.; Halage, A.A. Prevalence and risk factors associated with tungiasis in Mayuge district, Eastern Uganda. The Pan African medical journal 2016, 24.	The prevalence of tungiasis was 22.5%. However, a big percentage 41.5% of households were reported to have had *T. penetrans* in the previous month while 49.5% had *T. penetrans* for more than one month. Majority (90.5%) of the participants used a pin, needle, or thorn to remove sand flea from infected body parts.
34.7279	0.13321	Kenya	Nyangacha, R.M.; Odongo, D.; Oyieke, F.; Bii, C.; Muniu, E.; Chasia, S.; Ochwoto, M. Spatial distribution, prevalence and potential risk factors of Tungiasis in Vihiga County, Kenya. PLoS neglected tropical diseases 2019, 13, e0007244.	The occurrence of tungiasis was associated with low economic status (like a monthly income of Ksh ≤ 1000 (adjusted odds ratio 27.85; 95% CI: 4.13–187.59), earthen floor (0.36; 0.13–1.024) and lack of toilet facilities (4.27; 0.82–22.34), age of participant ≤14 (27.414; 10.02–74.99), no regular use of closed footwear (1.98; 0.987–3.97) and common resting place inside the house (1.93; 0.96–3.89).
37.0168	-0.9307	Kenya	Ruttoh, S.K.; Ochieng'Omondi, D.; Wanyama, N.I. Tunga penetrans A Silent Setback to Development in Kenya. Journal of Environmental Science and Engineering. B 2012, 1.	As a country, the Jigger menace is frustrating Kenya's attempts to achieve the Millennium Development Goal on poverty reduction by the year 2015, as well as reducing the pace of national development.
34.769	-0.67	Kenya	Ruttoh, S.K.; Ochieng'Omondi, D.; Wanyama, N.I. Tunga penetrans A Silent Setback to Development in Kenya. Journal of Environmental Science and Engineering. B 2012, 1.	As a country, the Jigger menace is frustrating Kenya's attempts to achieve the Millennium Development Goal on poverty reduction by the year 2015, as well as reducing the pace of national development.
35.9039	-1.0188	Kenya	Ruttoh, S.K.; Ochieng'Omondi, D.; Wanyama, N.I. Tunga penetrans A Silent Setback to Development in Kenya. Journal of Environmental Science and Engineering. B 2012, 1.	As a country, the Jigger menace is frustrating Kenya's attempts to achieve the Millennium Development Goal on poverty reduction by the year 2015, as well as reducing the pace of national development.
38.6037	-1.7231	Kenya	Zablon, W. Tungiasis Risk Factors in Rural Community in Murang'a County, Kenya. 2017. Kenyatta University (Dissertation).	Out of the 334 households sampled, 6.9% had at least tungiasis at the time of the study with a total of 35 cases. Most (65.8%) of those affected were children and 60% were males. Most (83.3%) of them were living in temporary houses and had domestic animals especially chicken which they interacted with.
39.7293	-3.4957	Kenya	Mwai, et al. Factors Influencing Practices Towards Water, Sanitation and Hygiene with Occurrence of Tungiasis among pupils in schools with a feeding programme in Ganze sub-County, Kenya. Global Journal of Health Sciences 2018, 3, 1–23.	This study found that environmental, demographic, and behavioral factors significantly predict tungiasis infestation.
30.1325	-4.5871	Tanzania	Mazigo, H.D.; Behamana, E.; Zinga, M.; Heukelbach, J. Tungiasis infestation in Tanzania. The Journal of Infection in Developing Countries 2010, 4, 187–189.	A 19-year-old male with epilepsy and mental disability presented with ulcerated and inflamed toes. Clinical examination revealed the presence of approximately 810 embedded jigger fleas on the feet, and another 60 lesions on the hands.

38.2475	-4.9892	Tanzania	Mwakanyamale, J.G.; Towett, R.K.; Mtango, F.; Bundala, J.; Kisanga, F. Contributions of socio-economic and cultural factors in Tungiasis at Kwakombo village in Korogwe district, Tanzania. IMTU Medical Journal 2015, 6, 54–61.	Out of the sample of 720 300 (41.7%) had infestation with *Tunga penetrans*, the majority of whom 207(69%) were males. The most vulnerable age group was 5-14 years (47%) and majority (95%) live in houses that are roofed with grass, with muddy floors and muddy walls, 58% did not wear shoes.
36.5717	-8.8704	Tanzania	Jeffreys, M. Pulex penetrans: the jigger's arrival and spread in Africa. S Afr J Sci 1952, 48, 249–255.	Literature Review and Geographical Dictionary
3.12192	6.48766	Nigeria	Samuel Ugbomoiko, U.; Ariza, L.; Emmanuel Ofoezie, I.; Heukelbach, J. Risk Factors for Tungiasis in Nigeria: Identification of Targets for Effective Intervention. 2007, 1, e87, doi:10.1371/journal.pntd.0000087.	Data from 643 individuals (86.6% of the target population) were analyzed; 252 (42.5%) were infested with *T. penetrans*. The presence of pigs on the compounds (adjusted odds ratio=17.98; 95% confidence interval: 5.55–58.23), sand or clay floor inside houses (9.33; 5.06–17.19), and having the common resting place outside the house (7.14; 4.0–14.29) were the most important risk factors identified.
4.6399	6.57725	Nigeria	Samuel Ugbomoiko, U.; Ariza, L.; Emmanuel Ofoezie, I.; Heukelbach, J. Risk Factors for Tungiasis in Nigeria: Identification of Targets for Effective Intervention. 2007, 1, e87, doi:10.1371/journal.pntd.0000087.	Data from 643 individuals (86.6% of the target population) were analyzed; 252 (42.5%) were infested with *T. penetrans*. The presence of pigs on the compounds (adjusted odds ratio=17.98; 95% confidence interval: 5.55–58.23), sand or clay floor inside houses (9.33; 5.06–17.19), and having the common resting place outside the house (7.14; 4.0–14.29) were the most important risk factors identified.
4.55136	8.49192	Nigeria	Samuel Ugbomoiko, U.; Ariza, L.; Heukelbach, J. Parasites of importance for human health in Nigerian dogs: high prevalence and limited knowledge of pet owners. BMC VR 2008, 4, doi:https://doi.org/10.1186/1746-6148-4-49.	Data from 643 individuals (86.6% of the target population) were analyzed; 252 (42.5%) were infested with *T. penetrans*. The presence of pigs on the compounds (adjusted odds ratio=17.98; 95% confidence interval: 5.55–58.23), sand or clay floor inside houses (9.33; 5.06–17.19), and having the common resting place outside the house (7.14; 4.0–14.29) were the most important risk factors identified.
32.6409	-13.627	Zambia	Mulambya, N.L.; Sakubita, P.; Hamoonga, R.; Mulubwa, B.; Namafente, O.; Mutengo, M.; Yard, E. Tungiasis Outbreak Investigation In Masaiti District, Zambia. Health Press Zambia Bull 2018, 2, 8–16.	192 tungiasis case-patients were sampled from August to October of which 120 (63%) were male, constituting an attack rate of 16%. Of the 66 cases interviewed, majority (40%) were children aged 0-9 years of which 66% were males. Almost all (91%) lesions were located on the feet.
30.3835	-4.2281	Tanzania	Nájera Villagrana, S.M.; García Naranjo Santisteban, A. Tungiasis: a highly neglected disease among neglected diseases. Case series from Nduta refugee camp (Tanzania). Oxford medical case reports 2019, 2019, omz049.	Seven severe cases of tungiasis in children living in a refugee camp in Tanzania were identified. All of whom were treated with surgical extraction of the fleas. Refugee camps-particularly in sub-Saharan Africa where tungiasis is endemic-should be considered high-risk areas for the condition.

48.45	-17.367	Madagascar	Thielecke, M.; Raharimanga, V.; Rogier, C.; Stauss-Grabo, M.; Richard, V.; Feldmeier, H. Prevention of tungiasis and tungiasis-associated morbidity using the plant-based repellent Zanzarin: a randomized, controlled field study in rural Madagascar. PLO.	Although shoes were requested by the villagers and wearing shoes was encouraged by the investigators at the beginning of the study, the availability of shoes only marginally influenced the attack rate of female sand fleas.
13.3556	7.21557	Cameroon	Richardson, D.J.; Mangili, A.M. Infection with the Sand Flea Tunga penetrans (Tungiasis) in a Traveller Returning from Cameroon, Africa. JAAS 2016, 70.	Case report of tungiasis in a traveler from Cameroon.
10.3887	5.25694	Cameroon	Bourée, P.; Simeni Njonnou, R.; Takougang, I.; Kaptue, L. Tungiasis in Bangou (West Cameroon). MST 2012, 22, 440–443, doi:10.1684/mst.2013.0134.	The prevalence in this population was 32.7%, with infestation more frequent in males (P = 0.013), mostly in children aged 1 to 5 years.
6.93032	4.89277	Nigeria	Arene, F.O.I. The prevalence of sand flea (Tunga penetrans) among primary and post-primary school pupils in Choba area of the Niger Delta. PH 1984, 98, 282–283, doi:https://doi.org/10.1016/S0033-3506(84)80004-9.	Overall, 30·4% of the pupils were infected with the parasite. More male pupils (33·8%) than female pupils (27·1%) harbored the infection.
10.7911	6.42726	Cameroon	Collins, G.; McLeod, T.; Njilah Issac, K.; Lamnyam, C.; Ngarka, L.; Leo Njamnshi, N. Tungiasis: A Neglected Health Problem in Rural Cameroon. IJCRIMPH 2009, 1, 2–10.	1,151 individuals were examined, including 567 males (49%) and 584 females (51%). Of these, 53% were children (0-14 years), and 10% were elderly (60+ years). In total, 610 individuals (53%) were infested with Tunga penetrans. Prevalence was higher in males (59%) than in females (47%) (p=0.004).
12.9463	-5.2664	Congo DRC	Bentley, W.H. Pioneering on the Congo; Religious tract society: London, 1900; Vol. 1.	Ethnological study
13.2296	-7.8726	Angola	Monteiro, J.J. Angola and the river Congo; Macmillan and Company: London, 1876.	Ethnological study
5.626	6.339	Nigeria	Ukonu, B.A.; Eze, E.U. Pattern of Skin Diseases at University of Benin Teaching Hospital, Benin City, Edo State, South-South Nigeria: A 12 Month Prospective Study. GJHS 2012, 4, 148–157, doi:10.5539/gjhs.v4n3p148.	Out of 4786 patients, 755 (15.8%) were new patients. The new patients comprised 96 (12.7%) children patients (< 15 years) and 659 (83.7%) adult patients (>15years). The ages of the patients ranged from 2 weeks to 80 years and more than two-third were < 40 years. There were 354 males (46.9%) and 401 females (53.1%).
15.2851	-4.2852	Congo	Stanley, H.M. Through the dark continent: or, the sources of the Nile, around the Great Lakes of Equatorial Africa, and down the Livingstone River to the Atlantic Ocean.; Sampson, Low: London, 1889.	A non-fiction account of travels to Africa
49.1039	-13.087	Madagascar	Thielecke, M.; Raharimanga, V.; Stauss-Grabo, M.; Rogier, C.; Richard, V.; Feldmeier, H. Regression of severe tungiasis-associated morbidity after prevention of re-infestation: a case series from rural Madagascar. AJTMH 2013, 89, 932–936, doi:https:	Eight individuals with extremely severe tungiasis in rural Madagascar were identified. To prevent reinfestation, four individuals received solid shoes and four received a daily application of an herbal repellent effective against *Tunga penetrans*. Over a period of 10 weeks the feet were examined, and the severity of tungiasis-associated morbidity was measured. Within this period, the severity score for acute tungiasis decreased 41% in the shoe group and 89% in the repellent group.
-10.612	6.38256	Liberia	Hesse, P. Die Ausbreitung des Sandflohs in Afrika. Ein tiergeographischer Versuch. GZ 1899, 522–530.	Literature Review
39.3077	-6.1329	Tanzania	Heukelbach, J.; Ugbomoiko, U.S. Tungiasis in the past and present: A dire need for intervention. NJP 2007, 28, 1–5.	Editorial

13.3872	-8.8482	Angola	Dias, J.R. Famine and disease in the history of Angola c. 1830–1930. TJOAH 1981, 22, 349–378.	In Angola, climatic instability contributed to maintaining a precarious balance between food resources, population, and disease long before the nineteenth century.
-16.398	16.0601	Senegal	Barbot, J. A description of the Coasts of Guinea in Churchill's A Collection of Voyages and Travels; London, 1732.	A non-fiction account of travels to Africa
-1.5715	5.1551	Ghana	Hutton, W. A Voyage to Africa: Including a Narrative of an Embassy to One of the Interior Kingdoms, in the Year 1820; with Remarks on the Course and Termination of the Niger, and Other Principal Rivers in that Country.; Longman, Hurst, Rees, Orme, and	Ethnology and Geographical Dictionary
-9.9742	8.72596	Guinea	Mollien, G.T.; Bowdich, T.E. Travels in the Interior of Africa, to the Sources of the Senegal and Gambia performed by command of the French Government in 1818; London, 1820.	A non-fiction account of travels to Africa
9.61387	0.34263	Gabon	Monteiro, J.J. Angola and the river Congo; Macmillan and Company: London, 1876.	Ethnological study
-7.6942	4.45943	Liberia	Johnston, H. Liberia; Hutchinson & Company: London, 1906; Vol. 1.	A non-fiction account of travels to Africa
9.70312	4.14093	Cameroon	Bentley, W.H. Pioneering on the Congo; Religious tract society: London, 1900; Vol. 1.	Ethnological study
16.2303	-2.1591	Congo DRC	Johnston, H. The River Congo, from Its Mouth to Bolobo: A General Description of the Natural History and Anthropology of Its Western Basin; Sampson Low, Marston & Company, 1895.	Ethnological study
26.9695	-10.4	Congo DRC	Crawford, D. Thinking black: 22 years without a break in the long grass of Central Africa; Morgan and Scott: London, 1912.	A non-fiction account of travels to Africa
34.9941	-15.798	Malawi	Decle, L. Three years in savage Africa; Methuen & Company: London, 1898.	A non-fiction account of travels to Africa
34.8805	-19.812	Mozambique	Decle, L. Three years in savage Africa; Methuen & Company: London, 1898.	A non-fiction account of travels to Africa
29.4456	-25.687	South Africa	Spencer, H.A. Chigger Flea or" Chigoe" in the Transvaal. TMJ 1912, 8.	*T. penetrans* in South Africa
30.3841	-29.617	South Africa	Jeffreys, M. Pulex penetrans: the jigger's arrival and spread in Africa. S Afr J Sci 1952, 48, 249–255.	Literature Review and Geographical Dictionary
5.05649	11.7433	Nigeria	Mockler-Ferryman, A.F. British West Africa: Its Rise and Progress; Swan Sonnenshein, 1900.	Ethnological study
39.4794	-1.9515	Kenya	Mwangi, M.M. Factors influencing participation of stakeholders in eradication of jiggers: A case of Kandara sub county, Muranga county, Kenya. 2015.	The study found out that among the demographic characteristics, only age and education level influenced participation. However, social, economic, and political factors had a significant influence on stakeholder s participation in jigger eradication.
38.6037	-1.7231	Kenya	Zabron, W. Tungiasis Risk Factors in Rural Community in Murang'a County, Kenya. 2017. Kenyatta University (Dissertation).	Out of the 334 households sampled, 6.9% had at least tungiasis at the time of the study with a total of 35 cases. Most (65.8%) of those affected were children and 60% were males. Most (83.3%) of them were living in temporary houses and had domestic animals especially chicken which they interacted with.
6.66106	0.29171	Sao Tome and Principe	Pampiglione, S.; Trentini, M.; Gentili, F.M.; Mendes, J.; Pampiglione, C.; Rivasi, F. Tunga penetrans (Insecta: Siphonaptera) in pigs in Sao Tomé (Equatorial Africa): Epidemiological, clinical, morphological and histopathological aspects. Rev Elev Me	28 subjects out of 100 examined were affected by lesions associated with Tunga penetrans (Insecta: *Siphonaptera*) localized particularly in the legs, snout, and udders. In the latter the presence of fleas inside the tissues of the teats caused heavy economic damage due to inability to nurse the sucking-pigs, resulting in the death of whole litters.

2.94314	6.44789	Nigeria	Ade-Serrano, M.A.; Ejezie, G.C. Prevalence of tungiasis in Oto-Ijanikin village, Badagry, Lagos State, Nigeria. 1981, 75, 471–472.	Of 373 children aged between 6 and 14 years (204 boys, 169 girls) studied, 41.5% (90 boys and 65 girls) had jiggers (*Tunga penetrans*) embedded in their toes (usually the sides and the pulp rather than the dorsum or the soles of the feet). The prevalence declined with age by about 50%.
37.323	0.993	Kenya	Ruttoh, S.K.; Ochieng'Omondi, D.; Wanyama, N.I. Tunga penetrans A Silent Setback to Development in Kenya. Journal of Environmental Science and Engineering. B 2012, 1.	As a country, the Jigger menace is frustrating Kenya's attempts to achieve the Millennium Development Goal on poverty reduction by the year 2015, as well as reducing the pace of national development.
22.2585	4.05163	Congo DRC	Johnston, H. The River Congo, from Its Mouth to Bolobo: A General Description of the Natural History and Anthropology of Its Western Basin; Sampson Low, Marston & Company, 1895.	Ethnological study
33.879	-9.4824	Tanzania	Johnston, H. The River Congo, from Its Mouth to Bolobo: A General Description of the Natural History and Anthropology of Its Western Basin; Sampson Low, Marston & Company, 1895.	Ethnological study
29.1977	-5.9071	Congo DRC	Johnston, H. The River Congo, from Its Mouth to Bolobo: A General Description of the Natural History and Anthropology of Its Western Basin; Sampson Low, Marston & Company, 1895.	Ethnological study
28.9394	-1.9219	Congo DRC	Gibbons, A.S.H. Africa from south to north through Marotseland; J. Lane, 1904; Vol. 2.	A non-fiction account of travels to Africa
37.7592	6.86033	Ethiopia	Ramaswamy, V. Assessment of socioeconomic status and the prevalence of Tungiasis in Jimma and Wolaita Sodo, Ethiopia. Int J Intg Med Sci 2016, 3, 211–15.	High prevalence of tungiasis could be significantly associated with the low income, poor housing and low standard living conditions and poor health care behavior of the population (p value <0.001).
38.5067	6.75577	Ethiopia	Girma, M.; Astatkie, A.; Asnake, S. Prevalence and risk factors of tungiasis among children of Wensho district, southern Ethiopia. BMC infectious diseases 2018, 18, 456.	Two hundred fifteen (58.7%, 95% confidence interval [CI]: 53.7%, 63.8%) of the 366 children were infested with *Tunga penetrans*. Most lesions were localized in the feet and the distribution of the disease by sex was similar (57.4% among males and 60.3% among females).
40.1745	6.74956	Ethiopia	Gadisa, E.; Jote, K. Prevalence and factors associated with intestinal parasitic infection among under-five children in and around Haro Dumal Town, Bale Zone, Ethiopia. BMCP 2019, 385, 1–8, doi:https://doi.org/10.1186/s12887-019-1731-0.	Of the 561 total under-five children, 216 (38.5%) were found to be infected with intestinal parasites. Regarding risk factors, geo-phage [(AOR = 4.7; 95%CI: 2.0-10.4), P < 0.001], tungiasis [(AOR = 3.1; 95%CI: 1.1-6.6), P < 0.001], eating raw vegetable [(AOR = 1.3; 95%CI: 1.4-3.3), P < 0.001] were significantly associated with intestinal parasitic infections.
10.2478	-0.6996	Gabon	Reiss, F. Tungiasis in New York City. Archives of Dermatology 1966, 93, 404–407.	A case, probably the first to be reported from New York City, involved a recent arrival from equatorial Africa, whose clinical manifestations were typical of *Tunga penetrans* infestation.
33.6625	0.89228	Uganda	Sentongo, E.; Wabinga, H. Tungiasis presenting as a soft tissue oral lesion. BMC oral health 2014, 14, 112.	Tungiasis of the tongue was diagnosed after microscopic examination.
35.4779	-4.2044	Tanzania	Dassoni, F.; Polloni, I.; Margwe, S.B.; Veraldi, S. Tungiasis in northern Tanzania: a clinical report from Qameyu village, Babati District, Manyara Region. The Journal of Infection in Developing Countries 2014, 8, 1456–1460.	A total of 62 schoolchildren (38 males and 24 females), with ages ranging from 6 to 14 years, were examined. Sixty children were infested by T. penetrans. A total of 865 lesions were observed: 170 lesions were vital and 695 were non-vital.

36.7816	-1.3329	Kenya	Karuga, J. Factors contributing to prevalence of jigger infestation among community members of Mugumoini sublocation. Gatanga district Kenya Kenya Medical Training College 2013.	The study also recommended that: The government through the ministry of education should ensure funding the free primary education, issue scholarships and charge low interest on loans to scholars among other strategic measures that would ensure quality education for all its citizens. This will increase the literacy level and reduce the rate of unemployment.
30.2073	-4.5028	Tanzania	Mazigo, H.D.; Bahemana, E.; Dyegura, O.; Mnyone, L.L.; Kweka, E.J.; Zinga, M.; Konje, E.T.; Waihenya, R.; Heukelbach, J. Severe tungiasis in Western Tanzania: case series. Journal of public health in Africa 2011, 2.	A total of 435 lesions were recorded with patients presenting with >75 lesions and showed signs of intense acute and chronic inflammation. Superinfection of the lesions characterized by pustule formation, suppuration and ulceration were common. Loss of nails and walking difficulty was also observed.
34.5968	-0.0055	Kenya	Okoth, A.A. Morbidity, Risk Factors, and flea species responsible for Tungiasis in selected villages in Kisumu County, Kenya. Nairobi: Kenya: Kenyatta University 2015.	Participants were between 1 and 83 years old (females (52.8%) and males (47.2%)). In 39.7% of households, at least one case of tungiasis was found. The overall prevalence was 19.5%, peaking among 5-9 years old's (37.7%) and the elderly (60+ years) (43.8%).
34.3174	-8.3333	Tanzania	Proctor, E.M. Tunga penetrans Acquired while Traveling in Africa. Canadian Journal of Infectious Diseases 1970, 5.	Three cases of tungiasis acquired during travel are briefly described, and the biology of the jigger flea, *Tunga penetrans*, is reviewed.
35.7943	0.50151	Kenya	Alfred, K. Factors Associated with Jigger Infestation in Kituro, Baringo Central District; A rapid appraisal report, 2009.	Jigger infestation prevalence rate was 33.2% and the most affected age of pupils was 6-12 years. Nearly a half 48.8% of those infested by jigger had a cultural belief that jigger infestation is caused by dirt while 31.5% perceived jigger to be harmful. Over 50% of the respondents acknowledged that 1-2 children in the households were infested. 62.4% reported removing jigger using thorns and 11.7% reported the use of separation of individuals who were jigger infested.
34.7682	-0.6883	Kenya	Ruttoh, S.K.; Ochieng'Omondi, D.; Wanyama, N.I. Tunga penetrans A Silent Setback to Development in Kenya. Journal of Environmental Science and Engineering. B 2012, 1.	As a country, the Jigger menace is frustrating Kenya's attempts to achieve the Millennium Development Goal on poverty reduction by the year 2015, as well as reducing the pace of national development.
34.7552	-0.6585	Kenya	Ruttoh, S.K.; Ochieng'Omondi, D.; Wanyama, N.I. Tunga penetrans A Silent Setback to Development in Kenya. Journal of Environmental Science and Engineering. B 2012, 1.	As a country, the Jigger menace is frustrating Kenya's attempts to achieve the Millennium Development Goal on poverty reduction by the year 2015, as well as reducing the pace of national development.
34.2975	0.6317	Kenya	Ruttoh, S.K.; Ochieng'Omondi, D.; Wanyama, N.I. Tunga penetrans A Silent Setback to Development in Kenya. Journal of Environmental Science and Engineering. B 2012, 1.	As a country, the Jigger menace is frustrating Kenya's attempts to achieve the Millennium Development Goal on poverty reduction by the

				year 2015, as well as reducing the pace of national development.
33.6512	0.50519	Uganda	Sentongo, E.; Wabinga, H. Tungiasis presenting as a soft tissue oral lesion. BMC oral health 2014, 14, 112.	Tungiasis of the tongue was diagnosed after microscopic examination.
35.418	-0.2099	Kenya	Waruguru, C.; Mwaniki, P.; Karama, M.; Muthami, L. Prevalence of tungiasis and its associated factors among residents of Kipkelion west sub-county; Kericho county, Kenya. Int J Health Sci Res 2015, 5, 434–45.	Two hundred fifteen (58.7%, 95% confidence interval [CI]: 53.7%, 63.8%) of the 366 children were infested with *Tunga penetrans*. Most lesions were localized in the feet and the distribution of the disease by sex was similar (57.4% among males and 60.3% among females).
39.8792	-3.5361	Kenya	Weise, S.; Feldmeier, H.; Larson, L.; Mambo, B. Household-related risk factors of tungiasis and severe disease in Kilifi County, Kenya. TMIH 2015.	The overall prevalence of tungiasis was 25.0% (95% CI 22.4-27.5%). Age-specific prevalence followed an S-shaped curve, peaking in the under-15-year-old group. In 42.5% of the households at least one individual had tungiasis. 15.1% of patients were severely infected (≥ 30 lesions).
40.1172	-3.2201	Kenya	Ruttoh, S.K.; Ochieng'Omondi, D.; Wanyama, N.I. Tunga penetrans A Silent Setback to Development in Kenya. Journal of Environmental Science and Engineering. B 2012, 1.	As a country, the Jigger menace is frustrating Kenya's attempts to achieve the Millennium Development Goal on poverty reduction by the year 2015, as well as reducing the pace of national development.
-7.028	17.3	Mauritania	Lugard, Lady A Tropical Dependency; London, 1905	Ethnological study
